# Role of TLR4 in Enteric Glia Response to *Clostridioides Difficile* Toxins: Insights From In Vivo and In Vitro Studies

**DOI:** 10.1111/jcmm.70943

**Published:** 2025-11-19

**Authors:** Maria Lucianny Lima Barbosa, Deiziane Viana da Silva Costa, Dvison Melo de Pacífico, Conceição da Silva Martins Rebouças, Cirle Alcantara Warren, Renata Ferreira Carvalho de Leitão, Gerly Anne de Castro Brito

**Affiliations:** ^1^ Center for Studies in Microscopy and Image Processing (NEMPI), Department of Morphology Federal University of Ceará Fortaleza Brazil; ^2^ Division of Infectious Diseases and International Health University of Virginia Charlottesville Virginia USA

**Keywords:** *Clostridioides difficile*, *Clostridium difficile*, enteric glia, toll‐like receptors, toxins

## Abstract

*Clostridioides difficile* ( *C. difficile* ) is a Gram‐positive anaerobic bacillus that causes intestinal disorders. Toll‐like receptor 4 (TLR4) plays a key role in innate immunity. This study examines the role of TLR4 in the response to *C. difficile* toxins, which induce cell death and inflammatory responses in enteric glial cells (EGCs). Male C57BL/6 mice were infected with *C. difficile* , and cecum samples were analysed 3 days post‐infection for TLR4 expression. In vitro, EGCs were exposed to *C. difficile* toxins with or without C34, a TLR4 antagonist, or pre‐exposed to TLR4‐specific 21‐nt small interfering RNAs (siRNA). TLR4 expression was assessed by immunocytochemistry, immunofluorescence, qPCR, and Western blotting. NFκB p65, TNF‐α, IL‐6, cleaved caspase‐3, and phosphatidylserine binding to annexin‐V were evaluated. TLR4 expression increased in infected intestinal tissue and toxin‐exposed EGCs. TLR4 antagonist or TLR4 knockdown reduced NFκB p65 nuclear translocation and TNF‐α expression but did not affect *IL‐6* upregulation. Additionally, TLR4 antagonist or TLR4 knockdown mitigated toxin‐induced cell death, as shown by decreased cleaved caspase‐3 and phosphatidylserine binding. These findings suggest that TLR4 contributes to *C. difficile* pathogenesis and that its inhibition reduces inflammation and prevents cell death in EGCs.

**Abbreviations**
C34: TLR4 antagonist
CDI: *Clostridioides difficile* infection
EGCs: Enteric glial cells
IFN: Interferon
IL6: Interleukin 6
LPS: lipopolysaccharide
MyD88: myeloid differentiation factor 88
NFκB p65: Nuclear factor kappa B subunit p65
qPCR: Quantitative real‐time PCR
TcdA: *Clostridioides difficile* toxin A
TcdB: *Clostridioides difficile* toxin B
TLR: Toll‐like receptors
TNF‐α: Tumour Necrosis Factor‐alpha

## 1 Introduction

*Clostridioides difficile* (formerly *Clostridium difficile* ) is a Gram‐positive bacterium that forms spores and is widely distributed in the intestinal tract of humans, animals, and the environment [1]. This anaerobic bacterium can cause a range of disorders, from mild diarrhoea to severe colitis and life‐threatening complications, such as pseudomembranous colitis, toxic megacolon, and bowel perforation [2, 3]. *Clostridioides difficile* infection (CDI) is a significant cause of healthcare‐associated diarrhoea in many countries and is associated with prolonged hospitalisation and higher mortality rates [4]. A prior study conducted by our group revealed a 48% infection positivity rate within a Brazilian cancer hospital [5]. Additionally, CDI has a substantial economic impact on healthcare systems and patients, costing over $1 billion annually in the United States alone [6, 7].

The bacterium produces three toxins, the primary virulence factors in CDI. These toxins are known as toxin A (TcdA), toxin B (TcdB), and *C. difficile* transferase (CDT), also referred to as a binary toxin. Clinical symptoms have been linked to these toxins [2]. TcdA and TcdB function as glucosyltransferases upon expression and secretion within the colon. They bind to host cell receptors, subsequently undergoing endocytosis by host cells. Once intracellular, these toxins inactivate Rho‐family GTPases through glucosylation. This inactivation disrupts the host cytoskeleton, leading to the accelerated breakdown of the epithelial barrier function in the colon. Consequently, inflammation, tissue damage, and diarrhoea are promoted [8]. Due to their capability to disrupt the intestinal epithelial barrier, *C. difficile* toxins can also affect underlying cells, inducing cytopathic and cytotoxic effects on enteric glial cells (EGCs) [9, 10, 11].

EGCs are important cellular components of the enteric nervous system (ENS), which is involved in regulating important gut functions such as motility and secretion. Persistent GI dysmotility, which is potentially associated with persistent damage to ENS cells, has been reported post‐CDI [12]. Understanding the mechanisms involved in EGCs' response to *C. difficile* toxins is crucial for better understanding how to prevent GI dysmotility postinfection.

The EGCs participate in host‐bacteria interactions, as demonstrated in an in vitro exposure model to enteroinvasive *Escherichia coli* or *Lactobacillus paracasei* ssp. *paracasei* F19. In this model, pathogenic and probiotic bacteria demonstrated distinct capabilities in modulating the expression of Toll‐like receptors (TLRs) within these cells [13]. TLRs are established molecular regulators of the immune response, playing a crucial role in recognizing and responding to invading pathogens [14]. Interestingly, EGCs express TLR1‐5, 7, and 9 [13]. Among these receptors, toll‐like receptor 4 (TLR4) holds particular significance. It is a key member of innate immunity, mediating inflammatory responses by recognizing lipopolysaccharide (LPS) and other ligands [15].

Previous studies have provided compelling evidence suggesting a potential association between *C. difficile* and TLRs. Notably, TLR‐5 has been identified as capable of recognizing flagellar proteins of *C. difficile* [16], while the TLR4 receptor can identify the surface layer proteins of the bacteria [17]. Additionally, the inhibition of Rho proteins by toxin B has been shown to enhance the expression of cytokines by regulating TLR 2, 3, and 4 in astrocytes [18, 19]. In a preclinical model of necrotizing enterocolitis, loss of TLR4 in enteric glia prevented the loss of these cells and avoided GI dysmotility [20]. However, it is essential to highlight that the precise role of TLR4 in CDI, especially regarding EGC, remains incompletely understood and warrants further investigation.

Understanding the mechanisms through which *C. difficile* toxins operate and the participation of TLR4 in this process can potentially drive advancements in products and discoveries beneficial to human health [2, 14]. Thus, this research aims to elucidate the role of TLR4 in cell death and the inflammatory response promoted by *C. difficile* toxins in EGCs.

## 2 Materials and Methods

The list of materials used in all experiments is available in the Supporting Information (Table S1).

### 2.1 Mice

Male C57BL/6 Mice, 8 weeks old, were obtained from the Jackson Laboratory, Farmington, US. Only male mice were used in this study: (1) to minimise variabilities associated with the estrous cycle in females, which can influence immune response and gut microbiota composition [21]; (2) and because male mice exhibit consistent and reproducible responses to CDI, enabling clearer interpretation of experimental outcomes [22] the mice were housed in polypropylene cages lined with wood shavings, with bedding changes occurring twice a week. Throughout the experiments, the animals were maintained in consistent environmental conditions (temperature: 22°C ± 2°C, with air exhaustion, and a 12 h light/12 h dark cycle). They had unrestricted access to water and standard chow *Ad libitum*. The experimental protocol received approval from the ethics of animal experiments committee at the University of Virginia (Protocol Number 4096). All the experiments involving animals were performed at the University of Virginia by Dr. Cirle Warren and Deiziane Costa, who are part of this manuscript

### 2.2 *C. difficile* Inoculum Preparation

*C. difficile* stocks (10 μL) were streaked onto TCCFA agar plates: *C. difficile* agar base (Oxoid, CM0601B) supplemented with 1% taurocholate (Sigma‐Aldrich, 86339), defibrinated horse blood (LAMPIRE Biological Laboratories, 7233401500ML), and cycloserine‐cefoxitin (Oxoid, SR0096E). After 24 h of incubation at 37°C in an anaerobic chamber (Coy Laboratory Products), a single colony was transferred to chopped meat broth (Anaerobe systems, AS811) and incubated for 16 h at 37°C under anaerobic conditions. Two 1 mL aliquots of the resulting culture were centrifuged (11,180 g, 1 min) to pellet the bacteria. The supernatant was discarded and replaced with fresh chopped meat broth (1 mL), followed by centrifugation (11,180 g, 1 min). This washing step was repeated four times to eliminate extracellular *C. difficile* toxins. One vial was used to determine bacterial concentration via McFarland turbidity standard using a densitometer (Den‐1B, BioSan). The other was adjusted in chopped meat broth to 10^8^ colony form unit (CFU)/mL. From this, a 100 μL inoculum containing 10^5^ CFU was prepared for mouse infection.

### 2.3 *C. difficile* Infection Model

**FIGURE 1:**
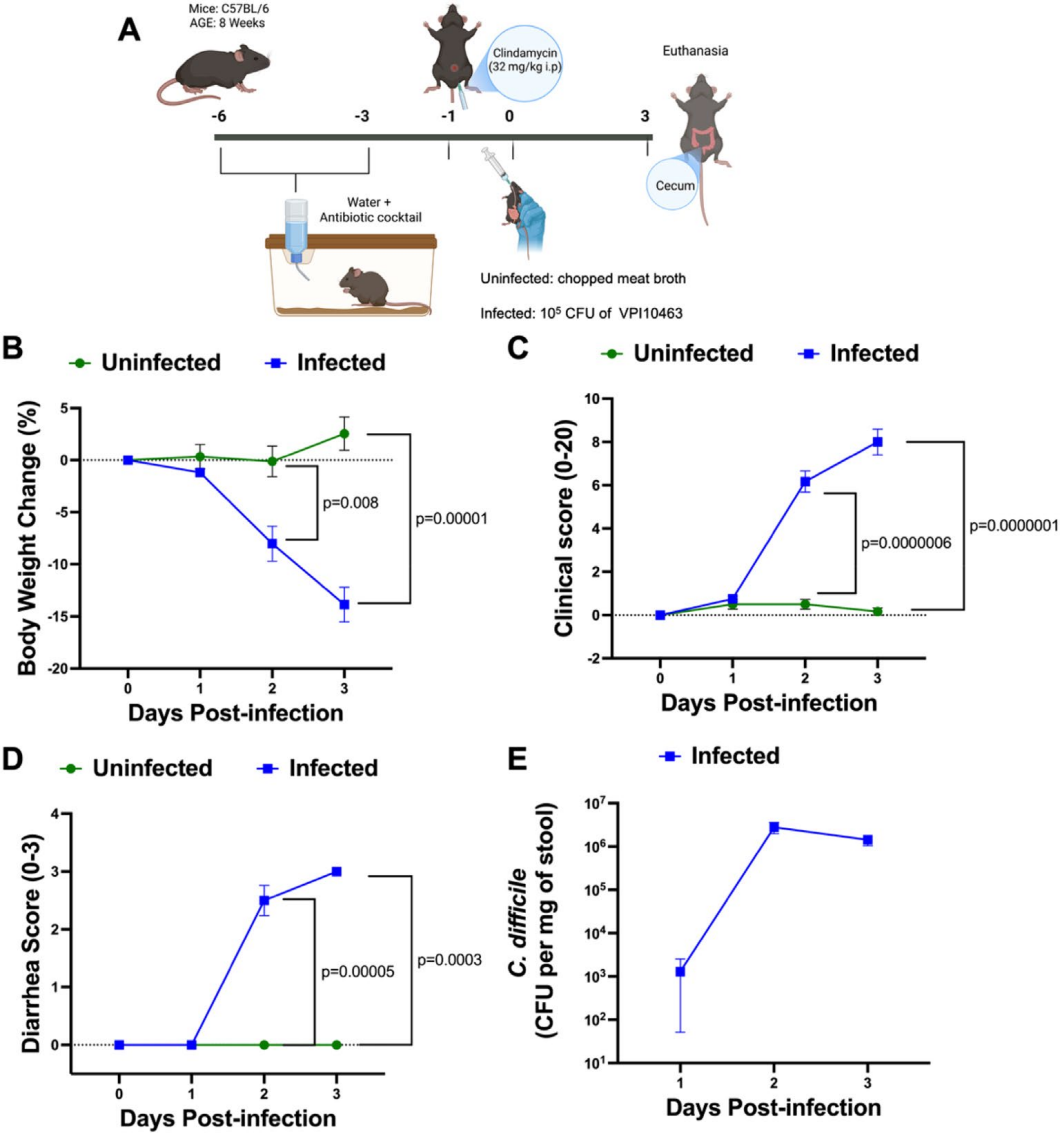
CDI model and disease course. (A) *C. difficile* infection model. (B) Body weight changes, (C) Clinical score (based on eyes, coat, posture, activity, diarrhoea, and body weight loss), and (D) Diarrhoea score in uninfected and infected mice. (E) *C. difficile* shedding in the stools of infected mice. (B‐E) The data represent the mean ± standard error of the mean (SEM). (*n* = 6). Multiple *t*‐test was used.

C57BL/6 mice (*n* = 6) underwent a three‐day antibiotic treatment in their drinking water, employing a previously described method [11, 22]. Following a one‐day antibiotic‐free period, an intraperitoneal injection of clindamycin (32 mg per kg) was administered one day before the *C. difficile* challenge. Subsequently, an oral gavage of 10^5^ CFU (in 100 μL of chopped meat broth, a pre‐reduced medium) containing the vegetative *C. difficile* strain VPI10463 (ATCC 43255, *tcdA* + *tcdB* + *cdtA* *‐* *cdtB*‐) was conducted (Figure 1A). Control mice received 100 μL of chopped meat broth. Mice were blinded evaluated daily to determine clinical score [23] and diarrhoea score [22] as previously described, as well as to determine *C. difficile* shedding as previously determined [22]. Animals were euthanized 3 days post‐infection utilizing tribromoethanol. Segments of cecum were collected and prepared accordingly for histological processing and analysis.

### 2.4 Immunohistochemistry

Sections (4 μm thick) were prepared from paraffin‐embedded mouse intestinal tissues. Following deparaffinisation, antigen retrieval was performed by incubating the slides in citrate buffer Target retrieval solution at low pH (K8005, Dako) for 20 min at 95°C in PTLink (PT10027, Dako). To reduce non‐specific binding, endogenous peroxidase was blocked with 3% H2O2 (Ab64218, Dako) for 10 min. The sections were then incubated overnight with an Anti‐TLR4 antibody (ab22048, Abcam, 1:400), followed by a 30‐min incubation with polymer HRP (K8000, Dako). The antibody‐binding sites were visualised by incubating the samples with diaminobenzidine–H2O2 (K3468, Dako) solution. The brown coloration identified positive staining, and the number of immunostaining cells was quantified using the ImageJ Software, counting cells with positive staining in ten different fields per slide (considering 1 slide per animal and four specimens per group) [24].

### 2.5 Cell Culture

The immortalised rat enteric glial cell (EGC) lineage PK060399egfr (ATCC CRL‐2690, VA, United States), a single rat enteric glial cell line available (37), was cultured in Eagle's medium modified by Dulbecco's DMEM (11965092, Gibco) supplemented with 10% fetal bovine serum (FBS A5256701, Gibco), 1% antibiotics (100 μg/mL penicillin and 100 μg/mL streptomycin, 15140–122, Gibco) and 1 mM sodium pyruvate (11360,070, Gibco). The cells were seeded in triplicate and maintained at 37°C in a humidified incubator under 5% CO_2_ for no more than 20 passages. EGCs were detached using 0.05% trypsin–EDTA (15090046, Gibco) for 5 min. To address the research questions of this study, EGCs were incubated with TcdA (50 ng/mL) and TcdB (1 ng/mL) for 18 h in all experiments. Both the concentrations and incubation time were selected based on previous studies [11, 22, 25], which demonstrated that these conditions effectively induce cellular responses without causing excessive nonspecific cytotoxicity.

In a set of experiments, C34 (50 μM), a potent and selective antagonist of TLR4, was added 1 h before toxins to study the role of this receptor in cell death and inflammatory responses induced by *C. difficile* toxins in enteric glia. The concentration of C34 used in this study was determined by using the MTT assay (Figure S1), which was performed as previously described [22].

To validate our pharmacological approach (TLR4 antagonist‐C34) findings, we knocked down TLR4 in enteric glia prior to *C. difficile* toxins (TcdA and TcdB) challenge by using a Silencer Select Pre‐designed TLR4‐specific 21‐nt small interfering RNAs (siRNA, Life technologies, s131044). Briefly, 10^4^ (in 100 μL of complete DMEM) and 4 × 10^4^ (in 500 μL of complete DMEM) cells were seeded in 96 well white plates and 4 well glass slides (Nunc, 154,526) respectively. After 24 h, cell media was replaced with DMEM supplemented with 10% FBS and and 1 mM sodium pyruvate. Then TLR4 siRNA and siRNA negative control (Life technologies, AM4611) were transfected into the cells by using lipofectamine RNAiMAX transfection reagent (Life technologies, 13,778–075), which was prepared according to the manufacturer's recommendation, for 6 h with media replacement and 24 h without media replacement (Figure S2A). Given that transfection for 6 h followed by media replacement did not affect the EGCs viability (Figure S2A) and was effective in knocking down TLR4 (Figure S2B and S2C), we performed all the experiments using this time of incubation. The final concentration of siRNA for experiments performed in 96 well plates and 4 well chamber was 1 pmol and 5 pmol respectively. The final amount of lipofectamine RNAiMAX used per well was 0.2 and 1 μL in 96 well plates and 4 well chamber respectively. After 24 h of transfection cells were incubated with *C. difficile* toxins at the same concentration mentioned above.

### 2.6 Immunocytochemistry Conducted on Enteric Glial Cells (EGCs)

Immunocytochemistry for TLR4 was performed, as previously described, by Veras‐Tinoco et al. [26]. EGCs at passage 14 were plated 5 × 10^4^ per well in a 24‐well plate. After 24 h of cultivation, cells were incubated with TcdA (50 ng/mL) or TcdB (1 ng/mL). After 18 h of incubation, cells were fixed with 4% paraformaldehyde‐PFA (43,388, Alfa Aesar) for 15 min and washed in 1% PBS. Then, antigenic retrieval was performed using citrate buffer pH 6.0 (K80004, Dako) for 30 min and endogenous peroxidase blockade (K80002, Dako) for 20 min. In the next step, the cells were incubated with the primary anti‐TLR4 antibody (482300, Invitrogen, 1:1000) for 20 min and with HRP polymer (K8000, Dako) for 30 min, being developed with the DAB chromogen (K3468, Dako) and counter‐stained with haematoxylin (K8008/K801, Dako). Coverslips were removed and placed on the slide with faramount (P36931, Dako). The percentage of TLR4 positive cells was determined by counting 100 cells per section from digital images captured from 5 areas within each section (*n* = 2–4), at × 400 magnification using ImageJ software. The number of immunopositive cells was divided by the total number of cells and multiplied by 100 to determine the percentage.

### 2.7 Immunofluorescence

EGCs at passage 15 were plated in 24‐well plates (4 × 10^4^ cells/well) or 4‐well glass slides (154526, Thermo Scientific), which were used for siRNA experiments. Following plating, C34 (50 μM), a TLR4 antagonist, was incubated, and after 1 h, TcdA (50 ng/mL) or TcdB (1 ng/mL) was added for an 18‐h incubation. In a set of experiments, cells were transfected with either a siRNA negative control or siRNA targeting TLR4, and after 24 h, they were incubated with *C. difficile* toxins for 18 h. Subsequently, cells were fixed in 4% paraformaldehyde (500 μL per well) (43488, Alfa Aesar) for 15 min. Afterward, cells were permeabilized in PBS (A59065BA, Thermo Fisher) with 0.025% Triton X100 (T8787, Sigma‐Aldrich) and 0.2% bovine serum albumin‐BSA (A2153‐100, Sigma‐Aldrich) for 15 min. Following permeabilization, cells were blocked with 0.25% Triton X‐100 (Sigma‐Aldrich) and 1% BSA in PBS at room temperature for 1 h, followed by washing samples with a washing solution. Subsequently, the cells were incubated with anti‐TLR4 (482300, Invitrogen, 1:500), nuclear factor kappa B (NFκB)‐p65 (ab16502, Abcam, 1:500), TNF‐α (ab6671, Abcam, 1:200) or anti‐cleaved caspase‐3 (AB3623, Sigma‐Aldrich, 1:100) overnight at 4°C. After three washes with washing buffer (0.01% Tween 20 in PBS), cells were incubated overnight with the secondary antibody AlexaFluor 594 (S11227, Invitrogen, 1:400) or AlexaFluor 488 (A11008, Thermo Scientific, 1:400) at room temperature for 2 h. Following a PBS wash, cells were mounted with ProLong Gold antifade reagent containing DAPI (P36931, Thermo Scientific). The samples were visualized using fluorescence microscopy (LM10, Zeiss Confocal). The immunofluorescence for TLR4, TNF‐α or cleaved caspase 3 was determined from digital images captured within each section (from four specimens per group) at × 400 magnification, using ImageJ software, as previously described [27]. The results are expressed as fluorescence intensity. The percentage of nuclear NFκB‐p65 positive cells was determined by counting 100 cells per section from digital images captured from various areas within each section (from four specimens per group), at × 400 magnification using ImageJ software. The number of immunopositive cells was divided by the total number of cells and then multiplied by 100 to determine the percentage [28].

### 2.8 Quantitative Real‐Time PCR (qPCR)

EGCs (6 × 10^5^ cells/well), at passage 15, were cultivated in 6‐well plates and treated with either TcdA (50 ng/mL) or TcdB (1 ng/mL). In some experiments, cells were incubated with C34 1h prior the challenge with toxins. The control group of cells received no treatment/vehicle. After incubation, total RNA extraction was performed using an RNeasy Plus Mini Kit (74104, Qiagen) on the QIAcube platform. RNA was quantified using a Qubit 3.0 fluorometer (life technologies), and its purity was assessed by the ratios of nucleic acids/proteins (260/280) and nucleic acids/other contaminants (260/230) obtained in the Nanodrop. Subsequently, the RNA underwent reverse transcription using a High‐Capacity cDNA Reverse Transcription Kit (4368814, Invitrogen) according to the manufacturer's protocol. qPCR amplification of *TLR4*, *IL‐6*, and glyceraldehyde 3‐phosphate dehydrogenase (*GAPDH*) in cell samples was performed using a StepOne apparatus (4376592, Applied Biosystems). Gene expression was calculated by the Livak & Schmittgen method (2–ΔΔCt). The coding sequence of the nucleotides obtained from the NCBI database (https://www.ncbi.nlm.nih.gov) and the OligoPerfect Primer Design Software (Thermo Fisher) was utilized for primer design. Primers are listed in Table S2.

### 2.9 Western Blotting

EGC (6 × 10^5^ cells/well), at passage 16, were cultured in six‐well plates and treated with either TcdA (50 ng/mL) or TcdB (1 ng/mL). After incubation, the supernatant was removed, and the cells were lysed using RIPA lysis buffer (89900, Thermo Scientific) containing EDTA and a free phosphatase protease inhibitor (P8340, Sigma‐Aldrich). The lysate underwent centrifugation at 13,000 rpm for 17 min at 4°C, and the supernatant was collected. Protein concentrations were determined using the bicinchoninic acid assay (23225, Thermo Scientific) according to the manufacturer's protocol. Forty micrograms of protein previously prepared with Laemmli sample buffer (1610747, Bio‐Rad) and β‐mercaptoethanol (1610710, Bio‐Rad) were denatured at 95°C for 5 min, separated on a 10% BIS‐Tris gel (NP0322BOX, Invitrogen), and transferred to PVDF membranes (1,620,177, Bio‐Rad) for 2 h. After blocking with 5% blocking solution (BioRad) at room temperature for 1 h, the membranes were incubated overnight with primary antibodies β‐actin 1:400 (Sigma‐Aldrich) and TLR4 1:100 (482300, Invitrogen). Subsequently, the membranes were incubated with secondary antibodies (anti‐rabbit 1:400) for 1 h and 30 min and then washed in Tris‐buffered saline containing 0.05% Tween 20‐TSB‐T (P1379, Sigma‐Aldrich) and incubated with Enhanced Chemiluminescence—ECL (1705060, BioRad). The chemiluminescence signal was detected using a ChemiDoc system (BioRad). Densitometric quantification of the bands was performed using ImageJ software.

### 2.10 Realtime‐Glo Annexin V Apoptosis Assay

Cell death was assessed using a live cell real‐time assay (Realtime‐Glo annexin V apoptosis assay, Promega, JA1000), following the manufacturer's instructions. EGCs (10^4^ cells/well), at passage 19, were seeded in white tissue culture‐treated 96‐well plates (Falcon, solid white bottom) and treated with TcdA (50 ng/mL) or TcdB (1 ng/mL) for 18 h in the presence or absence of a TLR4 receptor antagonist, C34 (50 μM), added 1 h before *C. difficile* toxin challenge. In a set of experiments, cells were transfected with either a siRNA negative control or siRNA targeting TLR4, and after 24 h, were incubated with *C. difficile* toxins for 18 h. Then, 200 μL of 2 × detection reagent (containing 2 μL of annexin NanoBit substrate, 2 μL of CaCl2, 2 μL of annexin V‐SmBit, and 2 μL of annexin V‐LgBit in 1000 μL of prewarmed supplemented DMEM) was added to each well. The cells were incubated at 37°C in a humidified incubator under 5% CO_2_. Luminescence was recorded using a Tecan multimode reader (Tecan), and the intrinsic reagent luminescence (no‐cell, no‐compound background control) was subtracted from the luminescence signals in the sample. The resulting values were then divided by the mean of control cells to obtain the relative luminescent units (RLUs).

### 2.11 Caspase 3/7 Activity

Caspase 3/7 activity was assessed using a luminescence assay (Caspase‐Glo 3/7 assay system, Promega, G8091), following the manufacturer's instructions. EGCs (10^4^ cells/well), at passage 20, were seeded in white tissue culture‐treated 96‐well plates (Falcon, solid white bottom) and transfected with siRNA negative control or siRNA TLR4. After 24 h, they were incubated with *C. difficile* toxins for 18 h. Then, 100 μL of Caspase‐Glo 3/7 reagent was added to each well, and the cells were mixed and incubated for 2 h at room temperature. Luminescence was recorded using a Tecan multimode reader (Tecan), and the intrinsic reagent luminescence (no‐cell, no‐compound background control) was subtracted from the luminescence signals in the sample. The resulting values were then divided by the mean of control cells to obtain the relative luminescent units (RLUs).

### 2.12 Statistical Analysis

The following tests were employed for statistical analysis between groups: the Shapiro–Wilk normality test to determine whether the data is parametric, a one‐way ANOVA analysis followed by Tukey's multiple comparison post‐test for parametric data. In addition, unpaired t‐test was applied to compare two groups. In cases where the data did not follow a normal distribution, the Kruskal‐Wallis test with Dunns post‐test was used. The confidence interval was set at 95%. Statistical analysis was performed using GradPad Prism software, version 8.0. All results are presented as mean ± standard error of the mean (SEM).

## 3 Results

### 3.1 *C. difficile* Infection Increases TLR4 Expression in the Cecum of Mice at the Peak of Clinical Symptoms

**FIGURE 2:**
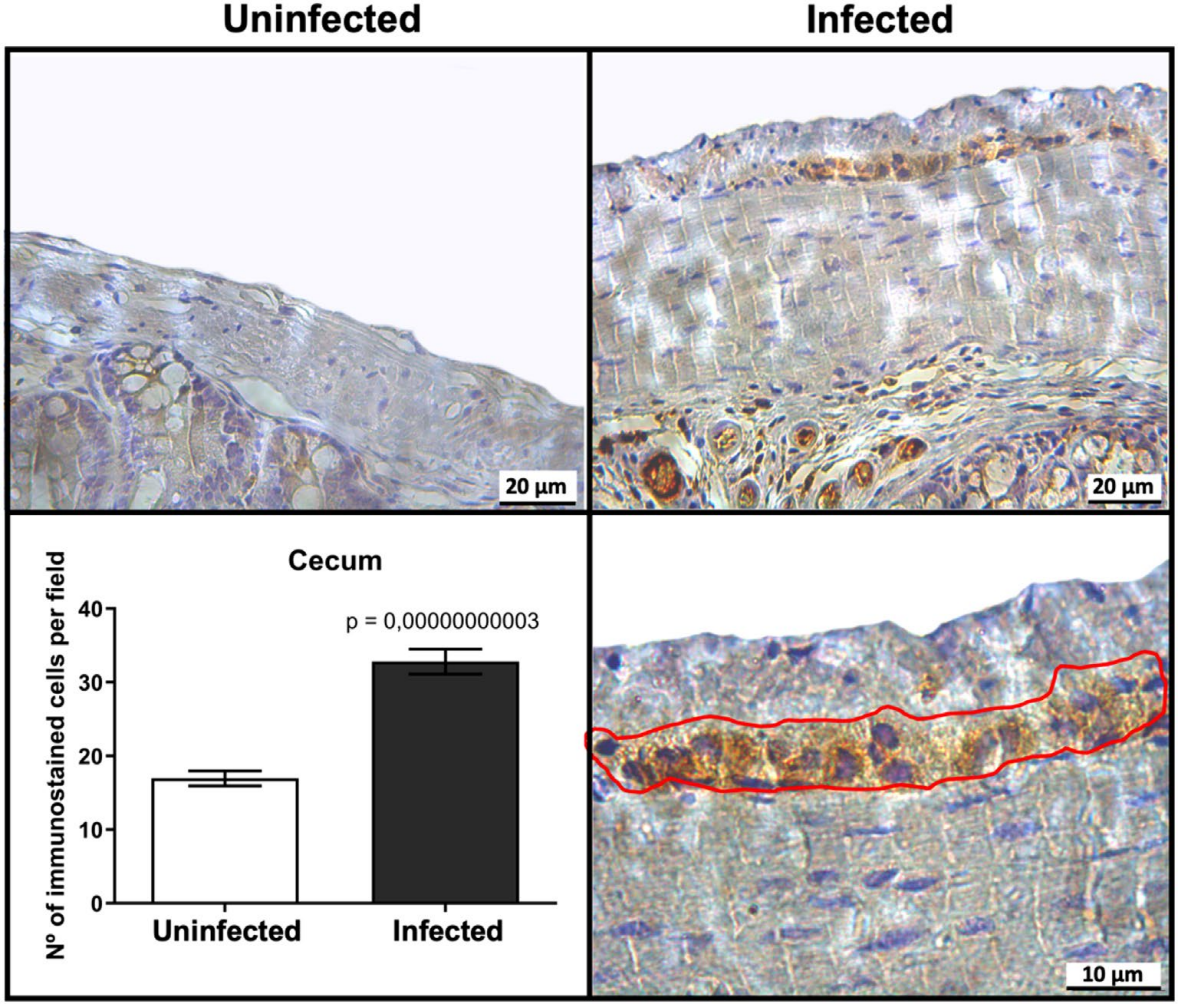
*C. difficile* infection increased the number of TLR4 immunostained cells in vivo. Immunohistochemistry photomicrographs illustrating cecum section tissue integrity in the control group (*n* = 4) and the infection group (*n* = 4) displaying immunostained cells in the myenteric plexus (highlighted by the red line), scale bars: 20 μm in the superior image and ten μm in the inferior image. The quantification of immunostained cells per field was performed in 10 fields for each animal, and the data are presented as the mean ± SEM. Two different experiments were performed to confirm these findings.

To better understand the distribution of TLR4 in cecum tissues, which is the most affected intestinal segment during mouse infection [22], at the peak of clinical symptoms (Figure 1B–E), we performed immunohistochemistry. *C. difficile* infection significantly increased TLR4 expression in the mouse cecum (*p* = 0.00000000003, Figure 2) compared to uninfected mice. A greater number of TLR4‐positive cells was observed in the submucosa and myenteric plexus (located in the muscular layer) compared to the control group (uninfected mice); however, it is important to note that the immunoexpression is not specific for glial cells. The myenteric plexus contains both glial cells and neurons. To specifically investigate glial cells, we next performed in vitro experiments.

### 3.2 *C. difficile* Toxins Induce Upregulation of the TLR4 Gene and Protein Expression in EGC in Vitro

**FIGURE 3:**
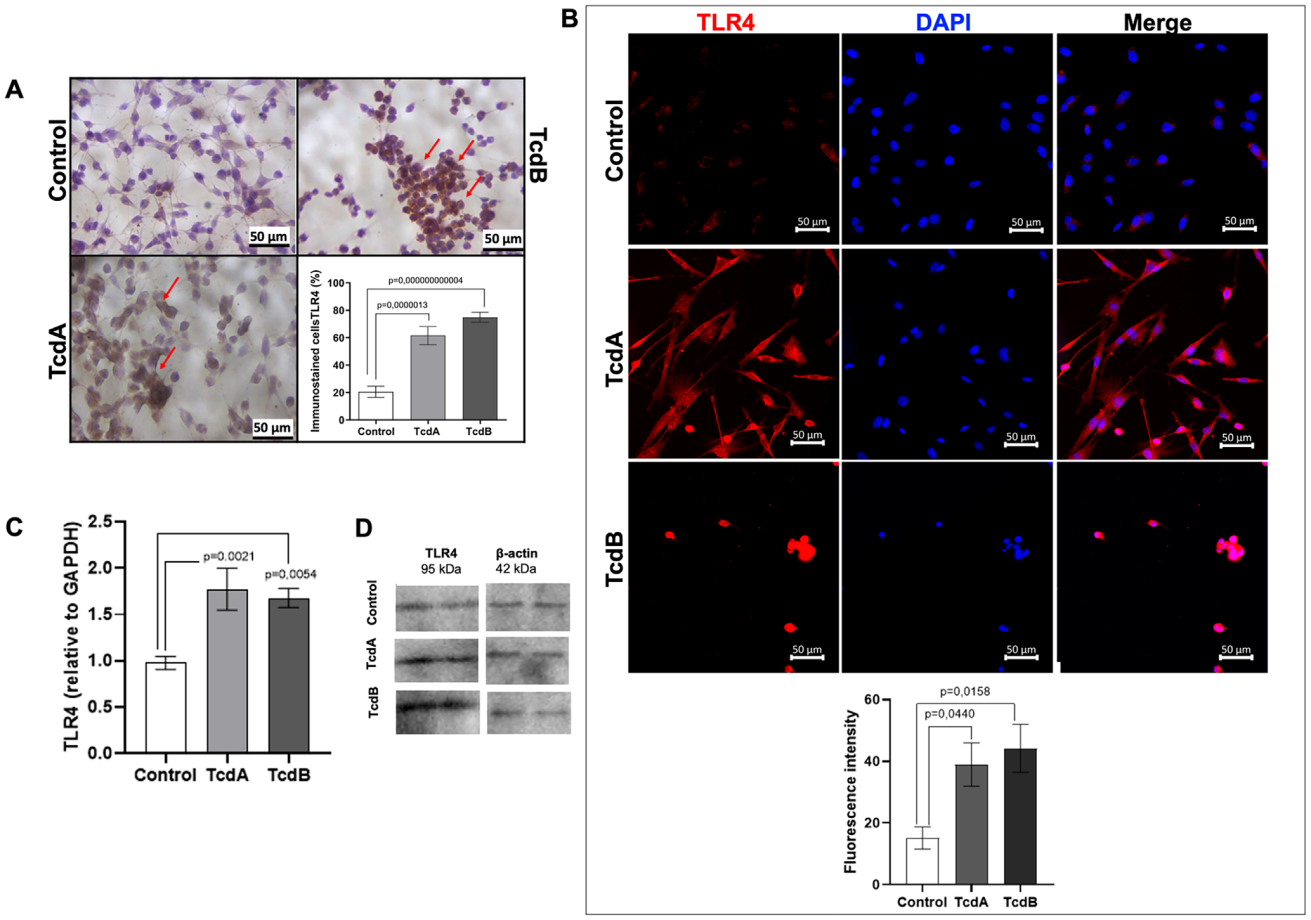
*C. difficile* Toxins A (TcdA) and B (TcdB) induce increased TLR4 gene and protein expression in EGCs. A. Arrows are pointing to TLR4‐immunopositive cells, stained in brown. The control group exhibited few immunostained cells, while EGCs challenged with TcdA (50 ng/mL) or TcdB (1 ng/mL) showed a notable increase in TLR4‐immunoexpression (*n* = 2–4). The plotted data come from 5 fields per coverslip, totaling 10 to 20 observations per group. Scale bars: 50 μm. B. Photomicrographs of TLR4 (red) immunostaining and DAPI (blue) nuclear staining in EGCs exposed to TcdA (50 ng/mL) or TcdB (1 ng/mL) for 18 h and measurement of TLR4 fluorescence intensity in EGCs using ImageJ software. Merge represents the combined image of TLR‐4 fluorescence and nuclear staining. Scale bars: 50 μm. The percentage of fluorescence intensity was higher in the TcdA and TcdB groups (*n* = 4) than in the control group (*n* = 5). C. TcdA and TcB induced upregulation in *TLR4* gene expression analysed by qPCR assay (qPCR) (*n* = 10–11). D. Representative Western Blot images demonstrating the increase in TLR4 protein expression induced by *C. difficile* toxins. β‐Actin was used as a housekeeping protein. The data represent the mean ± SEM. One‐way ANOVA followed by the Tukey's test was used.

Given that EGCs are one of the cellular components of the myenteric plexus, which is an area exhibiting higher TLR4‐positive staining, we next evaluated whether *C. difficile* toxins could directly affect the expression of TLR4 in these cells using our well‐established in vitro model [11, 22, 25, 27]. EGCs exposed to TcdA (50 ng/mL) or TcdB (1 ng/mL) for 18 h displayed a statistically significant increase in TLR4‐immunoexpression, visualised through brown stained EGC cells (Figure 3A, *n* = 2–4) and higher fluorescence intensity in these cells (Figure 3B, *n* = 4–5). Confirming these findings, exposure to *C. difficile* toxins increased TLR4 gene expression (Figure 3C, *n* = 10–11) and protein expression (Figure 3D, *n* = 3).

### 3.3 TLR4 Antagonist Decreased NFκB‐p65 Nuclear Translocation Induced by *C. difficile* Toxins in EGCs

**FIGURE 4:**
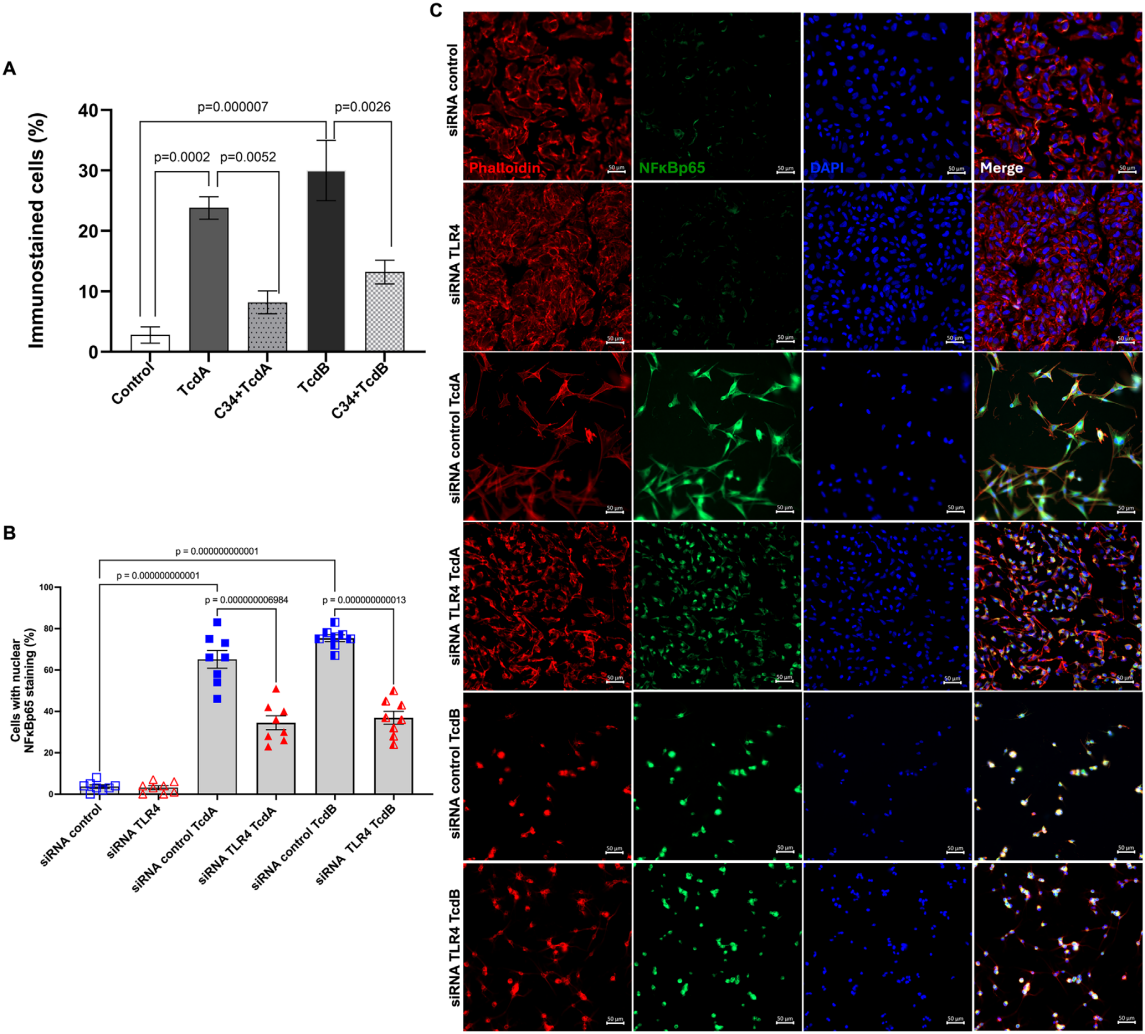
TLR4 Antagonist decreases NFκB‐p65 nuclear translocation induced by TcdA and TcdB. (A) The percentage of NFκB‐p65 positive was evaluated for every 100 cells counted (*n* = 5), focusing on the hot areas of each group. EGCs exposed to TcdA (50 ng/mL) or TcdB (1 ng/mL) after 18 h of incubation in the presence or absence of C34 (50 μM). (B) The percentage of cells with nuclear NFκB‐p65 positive staining (*n* = 8) in EGCs transfected with siRNA control or siRNA TLR4 and exposed to TcdA (50 ng/mL) or TcdB (1 ng/mL) for 18 h. A,B. The data are presented as the mean ± SEM. The one‐way ANOVA test was followed by Tukey's test; the *p*‐value is represented in the graph. (C) Representative photomicrographs illustrating a marker of Actin filaments, phalloidin (red), NFκB‐p65 (green) immunostaining, and DAPI (blue) nuclear staining in EGCs transfected with siRNA control or siRNA TLR4 and exposed to TcdA (50 ng/mL) or TcdB (1 ng/mL) for 18 h. Merge represents the combined image of phalloidin, NFκB‐p65 and nuclear staining. Scale bars: 50 μm.

Given that the transcription factor NFκB plays a crucial role in the inflammatory response mediated by *C. difficile* toxins in enteric glia [22], we investigated the role of TLR4 in modulating its nuclear translocation in these cells. For that, we pretreated EGCs with a TLR4‐specific antagonist (C34) before incubating them with TcdA or TcdB. Photomicrographs illustrate the localization of NFκB‐p65 (Figure S3), indicating its augmented translocation to the nucleus in response to *C. difficile* toxins. We found that C34 (50 μM) decreased the percentage of NFκB‐p65 translocation to the nucleus induced by TcdA (*p* = 0.0052) and TcdB (*p* = 0.0026) in EGCs (Figure 4A, *n* = 5). Using an siRNA‐mediated knockdown approach, we demonstrated that silencing TLR4 in EGCs exposed to TcdA or TcdB significantly reduces NFκB‐p65 nuclear translocation in response to *C. difficile* toxins (Figure 4B,C).

### 3.4 TLR4 Antagonist Modulates TNF‐α Synthesis, but Not *IL‐6* Gene Expression, in EGCs Challenged With *C. difficile* Toxins

**FIGURE 5:**
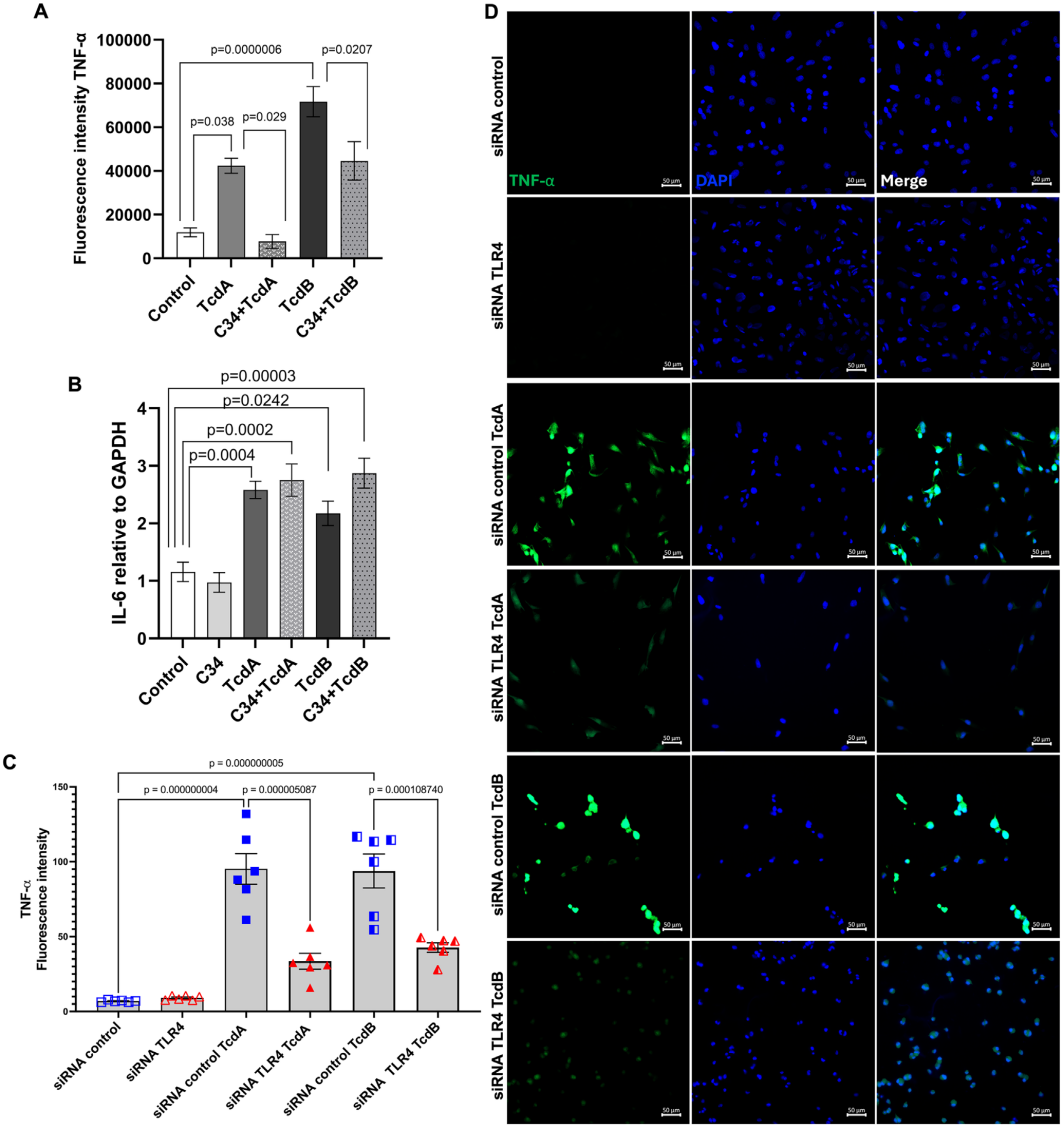
TLR4 antagonist prevents increased TNF‐α expression in EGCs induced by TcdA and TcdB. A. The intensity of TNF‐α staining (the green fluorescence) was determined with ImageJ in the hot areas (*n* = 4–8), and B. *IL‐6* gene expression (*n* = 5–6) was measured by qPCR in EGCs exposed to TcdA (50 ng/mL) or TcdB (1 ng/mL) after 18 h of incubation in the presence or absence of C34 (50 μM). C. The intensity of TNF‐α staining in EGCs transfected with siRNA control or siRNA TLR4 and exposed to TcdA (50 ng/mL) or TcdB (1 ng/mL) for 18 h. A‐C. The data are presented as the mean ± SEM, and statistical analysis was performed using the one‐way ANOVA test followed by Tukey's test. The corresponding *p*‐values are indicated in the graph. D. Representative photomicrographs illustrating TNF‐α (green) immunostaining and DAPI (blue) nuclear staining in EGCs transfected with siRNA control or siRNA TLR4 and exposed to TcdA (50 ng/mL) or TcdB (1 ng/mL) for 18 h. Merge represents the combined image of TNF‐α and nuclear staining. Scale bars: 50 μm.

TNF‐α and IL‐6 are well‐known downstream targets of NFκB activation [15]. Our findings suggest that the TLR4 antagonist (C34) effectively inhibits the increased TNF‐α protein expression induced by both TcdA and TcdB (*n* = 4–8) (Figures S4 and S5A). However, C34 (50 μM) did not prevent the TcdA and TcdB‐induced upregulation of *IL‐6* gene expression in EGCs (Figure 5B). Silencing TLR4 in EGCs exposed to TcdA or TcdB significantly reduced TNF‐α protein expression in response to TcdA or TcdB (Figure 5C,D), supporting the results obtained with the pharmacological approach.

### 3.5 TLR4 Antagonist Decreases EGC Death Induced by *C. difficile* Toxins

**FIGURE 6:**
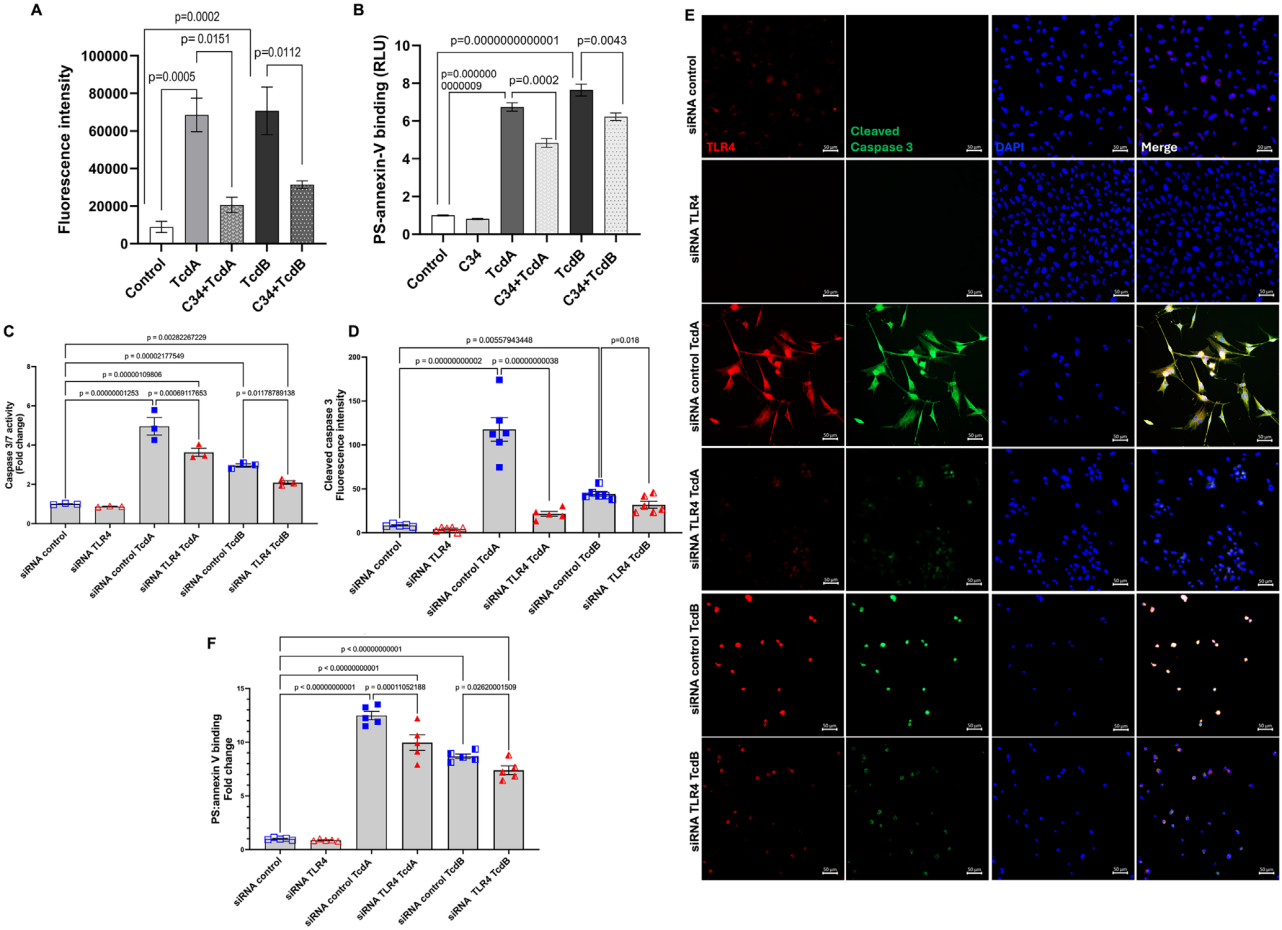
TLR4 antagonist decreases cellular death induced by TcdA and TcdB in enteric glia. A. Cleaved caspase‐3 staining was evaluated in five fields per group (*n* = 3–6) in EGCs incubated for 18 h with TcdA (50 ng/mL) or TcdB (1 ng/mL) in the presence or absence of C34 (50 μM), a TLR4 antagonist, which was added 1 h before toxin challenge. B. Cell death (*n* = 3–5) was analysed by RealTime‐Glo annexin V apoptosis assay in EGCs incubated for 18 h with TcdA (50 ng/mL) or TcdB (1 ng/mL) in the presence or absence of C34 (50 μM). C. Caspase 3/7 activity and D. Cleaved caspase 3 staining in EGCs transfected with siRNA control or siRNA TLR4 and exposed to TcdA (50 ng/mL) or TcdB (1 ng/mL) for 18 h. E. Representative photomicrographs illustrating TLR4 (red) and cleaved caspase 3 (green) immunostaining, as well as DAPI (blue) nuclear staining in EGCs transfected with siRNA control or siRNA TLR4 and exposed to TcdA (50 ng/mL) or TcdB (1 ng/mL) for 18 h. Merge represents the combined image of TLR4, cleaved caspase 3 and nuclear staining. Scale bars: 50 μm. F. Cell death was analysed by RealTime‐Glo annexin V apoptosis assay in EGCs transfected with siRNA control or siRNA TLR4 and exposed to TcdA (50 ng/mL) or TcdB (1 ng/mL) for 18 h. A‐D and F [12]. The one‐way ANOVA test was used for statistical analysis, followed by the Tukey's test; The corresponding *p*‐values are indicated in the graph.

To investigate the role of the TLR4 receptor in cell death induced by *C. difficile* toxins, we examined the impact of C34 (50 μM), a TLR4 antagonist, on cleaved caspase‐3 immunostaining in EGCs and the binding of annexin and phosphatidylserine. Inhibition of TLR4 significantly reduced the immunostaining of cleaved caspase‐3 in EGCs challenged with *C. difficile* toxins (*p* < 0.0001, *n* = 3–6, Figures S5 and 6A). Furthermore, the pre‐incubation of EGCs with C34 (50 μM) effectively prevented cell death induced by TcdA (*p* = 0.0002) and TcdB (*p* = 0.0043), as evidenced by the analysis of phosphatidylserine–annexin V binding (Figure 6B, *n* = 3–5). Silencing TLR4 in EGCs significantly reduced caspase 3/7 activity (Figure 6C), immunostaining of cleaved caspase 3 (Figure 6D,E), and cell death (Figure 6F) in response to *C. difficile* toxins, supporting the results obtained with the pharmacological approach.

## 4 Discussion

Our study aimed to investigate the role of TLR4 in the death of EGCs induced by *C. difficile* toxin and the inflammatory response it triggers. A notable strength of this investigation lies in examining the impact of *C. difficile* toxins on EGCs. This area has been less explored than the predominant focus on intestinal epithelial and immune cells in existing research. EGCs are increasingly acknowledged as pivotal regulators of diverse intestinal functions, including motility, secretion, epithelial barrier integrity, and overall gut balance in healthy and diseased conditions. Undergoing a transition into a reactive glial phenotype in response to intestinal inflammation, these cells contribute to enteric gliosis [29]. Our recent studies have highlighted EGC reactivity in CDI [27], emphasising the impact of *C. difficile* toxins on these cells, thereby amplifying the inflammatory response [27]. However, further investigations into the potential influence of EGCs on CDI outcomes are needed.

The present study found a significant upregulation of TLR4 expression in cecum tissues from *C. difficile* ‐infected animals. Additionally, in our in vitro experiments, exposure to *C. difficile* toxins increased TLR4 expression in EGCs. Moreover, a TLR4 antagonist (C34) promoted the downregulation of inflammatory markers, specifically TNF‐α, and mitigated cell death induced by *C. difficile* toxins.

TLR receptors play a crucial role in innate immunity and are vital in gram‐positive and gram‐negative bacterial infections [30]. Increased TLR4 expression has been linked to several conditions, including hypoxia‐induced damage to intestinal barrier function and bacterial translocation [31], necrotizing enterocolitis [32], systemic inflammation induced by the chemotherapy drug doxorubicin [33], and neuroinflammation triggered by LPS [34]. Thus, upregulation of the TLR4 is a recurring phenomenon in inflammatory conditions.

In our study, increased TLR4 expression was detected in the submucosa and myenteric plexus of the cecum of *mice* infected with *C. difficile* . Lai et al. [30] also reported increased TLR4 protein expression in the large intestine and rectum of mice during CDI.

Histologically, the ENS is organised as plexuses, consisting of interconnected neurons and EGCs [12] *C. difficile* toxins disrupt the intestinal epithelial barrier and can affect underlying cells, including enterocytes, colonocytes, enteric neurons, and EGCs [9, 12]. Previous studies from our group have demonstrated the susceptibility of EGCs to TcdA and TcdB [11, 22]. However, further investigations are necessary to understand the mechanisms involved in this effect. Based on this, we explored the role of the TLR4 receptor in *C. difficile* toxin‐mediated cell death and inflammatory response in enteric glia.

The TLR4 signalling pathway is categorised into two pathways: a myeloid differentiation factor 88 (MyD88)‐dependent pathway, leading to the activation of NFκB and subsequent induction of inflammatory cytokines, such as TNF‐α and IL‐6, and a TRIF‐dependent pathway, responsible for inducing Type I interferon (IFN) along with inflammatory cytokines [35, 36, 37].

Here, we demonstrate that TLR4 is low‐expressed in EGCs under physiologic conditions. However, challenges with TcdA and TcdB resulted in an upregulation of TLR4 expression and subsequent activation, suggesting the involvement of this receptor in the pathogenesis of CDI.

Previous studies have shown that EGCs release nitric oxide through TLR4 upon activation, thereby releasing pro‐inflammatory cytokines that contribute to the exacerbation of gut inflammation [13]. Based on this, we hypothesized that modulating TLR4 could benefit CDI. In the present study, we explored the impact of TLR4 receptor inhibition on the response of EGCs to *C. difficile* toxins. This investigation covered various aspects, including the activation of NFκB, the expression of pro‐inflammatory cytokines (TNF‐α and IL‐6), and cell death.

NFκB activation involves the enzymatic degradation of the bound inhibitory protein, leading to the exposure of the nuclear localization signal (NLS) on p50 and p65. This enables the translocation of subunits from the cytoplasm to the nucleus [38]. Our study observed that the TLR4 antagonist (C34) or its knockdown using the siRNA approach decreased NFκB activation, as indicated by the reduced translocation of NFκB p65 to the nucleus.

CDI induces a systemic inflammatory cascade characterised by elevated levels of IL‐6 and TNF‐α [39]. Therefore, we aimed to investigate whether inhibiting TLR4 could prevent the upregulation of these cytokines induced by TcdA and TcdB. Our findings revealed that C34 reduced the *C. difficile* ‐induced increase in TNF‐α protein expression in EGCs but did not impact *IL‐6* gene expression in these cells. The literature has argued that the magnitude of IL‐6 upregulation in CDI is greater than that of TNF‐α [39], and IL‐6 can be modulated by various factors, including other receptors involved in the pathogenesis of CDI [11, 27]. In enteric glia challenged with *C. difficile* toxins, modulation of RAGE [22] and A2A adenosine receptor [27] has been shown to contribute significantly to the modulation of IL‐6. Hence, TLR4 inhibition alone may not be sufficient to prevent the upregulation of IL‐6 induced by *C. difficile* toxins in EGCs. Interestingly, IL‐6 is a pleiotropic cytokine with destructive and beneficial potentials and can even inhibit the apoptosis of neuronal cells after injury [40].

On the contrary, a study has demonstrated that EGCs exhibit increased susceptibility to TcdB‐induced apoptosis when triggered by TNF‐α and IFN‐γ [10]. Given that both TNF‐α and IFN‐γ can be released upon TLR4 activation, our focus shifted to exploring the impact of TLR4 inhibition on cell death. Interestingly, the TLR4 antagonist (C34) notably reduced the mortality of EGCs. This reduction was evidenced by diminished PS‐annexin V binding and caspase 3 activity. These findings strongly suggest the potential involvement of this receptor in facilitating the death of EGCs induced by *C. difficile* toxins. Notably, the role of TLR4 in cell death has been observed in other cell types, such as endothelial cells [41] and in the mouse brain [42]. In EGCs, TcdB demonstrated the ability to trigger three apoptotic pathways [10], among which the caspase pathway stands out. This pathway involves the activation of caspase‐3, caspase‐7 (effector caspases), caspase‐9, and PARP, all associated with increased apoptosis.

Our collected data present compelling evidence of TLR4's substantial involvement in CDI pathogenesis, showcasing its upregulation in the intestines of infected mice and EGCs. Moreover, our study emphasises the efficacy of the TLR4 inhibitor in reducing inflammatory markers and cell death. Previous studies have investigated TLR4 activation in cell types beyond EGCs following exposure to *C. difficile* components. Ryan et al. [17] demonstrated that *C. difficile* surface layer proteins (SLPs) activate NFκB signalling via TLR4 in bone marrow–derived dendritic cells, inducing dendritic cell maturation and secretion of IL‐12, TNF‐α, and IL‐10, as well as driving Th1/Th17 responses, including IFN‐γ and IL‐17. In vivo, TLR4‐deficient mice exhibited increased disease severity in response to *C. difficile* , highlighting the functional importance of TLR4 in host defence [17]. More recently, Noori et al. [43] reported that SlpA from selected *C. difficile* ribotypes variably downregulated the expression of tight junction–associated genes and increased TLR4 expression and pro‐inflammatory cytokine production in HT‐29 cells, a human colorectal adenocarcinoma cell line widely used as an in vitro model of intestinal epithelial cells [43]. These findings indicate that TLR4‐dependent signalling in response to *C. difficile* toxins or components is not restricted to EGCs, supporting the broader relevance of our observations in enteric glia. These findings have important clinical implications, particularly in light of the emerging epidemic and the diminishing efficacy of existing antimicrobial therapies for severe CDI.

Relying on immune‐based antimicrobial therapies, such as TLRs, could offer a promising new avenue for combating intestinal infections [30]. TLR4 antagonists, such as eritoran, which did not show success in phase III clinical trials for sepsis [44], and ApTOLL, which was safe and effective in reducing mortality in human subjects with acute ischemic stroke [45], have been previously tested. Although C34 has not yet been applied in human clinical trials, it has been effectively used in preclinical studies and in vitro studies [46] to understand the role of TLR4 in disease settings. Further studies are needed to understand better the optimal time point in the disease course and the target population for TLR4 use in CDI. Because TLR4 is expressed in multiple cell types, a significant challenge for therapeutic application is ensuring targeted delivery only to sites where inhibition is needed.

While our study provides valuable insights, its reliance on in vitro studies and samples from a preclinical mouse model may limit the direct applicability of the findings to human infections. Nevertheless, our study is pivotal in advancing and perpetuating research on the interplay between TLR4 receptors and *C. difficile* toxins in enteric glia, an important cell involved in gut motility and homeostasis. It encourages the exploration of other components within the TLR4 pathway, including the MyD88‐dependent pathway (MyD88/NFκB) and the MyD88‐independent pathway leading to interferons. Moreover, exploring diverse signalling pathways could benefit further investigation into the TLR4 inhibitor's impact on cell death. Future studies could be designed to delve deeper into this receptor's involvement and its clinical implications in the outcomes of CDI.

## Author Contributions

**Maria Lucianny Lima Barbosa:** data curation (equal), formal analysis (equal), investigation (equal), project administration (equal), writing – original draft (equal). **Deiziane Viana da Silva Costa:** formal analysis (equal), investigation (equal), project administration (equal), writing – original draft (equal). **Dvison Melo de Pacífico:** investigation (supporting). **Conceição da Silva Martins Rebouças:** data curation (equal), investigation (equal). **Cirle Alcantara Warren:** conceptualization (equal), formal analysis (equal). **Renata Ferreira Carvalho de Leitão:** writing – review and editing (lead). **Gerly Anne de Castro Brito:** conceptualization (equal), formal analysis (equal), funding acquisition (equal), supervision (lead), writing – original draft (equal).

## Ethics Statement

The experiments and protocols involving animals were approved by the Institutional Animal Care and Use Committee (IACUC) at the University of Virginia (protocol number: 4096, rodent model of *C. difficile* infection). This protocol is reviewed for additional procedures every 3 years.

## Conflicts of Interest

The authors declare no conflicts of interest.

## Supporting information


**Table S1:** List of materials used in this study
**Table S2:** Primer set used in this study.
**Figure S1:** jcmm70943‐sup‐0001‐supinfo.docx.
**Figure S2:** jcmm70943‐sup‐0001‐supinfo.docx.
**Figure S3:** jcmm70943‐sup‐0001‐supinfo.docx.
**Figure S4:** jcmm70943‐sup‐0001‐supinfo.docx.
**Figure S5:** jcmm70943‐sup‐0001‐supinfo.docx.

## Data Availability

Data available in article Supporting Information.
