# Supplementary material for: Role of TLR4 in Enteric Glia Response to Clostridioides Difficile Toxins: Insights From In Vivo and In Vitro Studies
Source: J Cell Mol Med. 2025 Nov 19;29(22):e70943. doi: 10.1111/jcmm.70943 (PMC12629862; doi:10.1111/jcmm.70943)
Supplement: Supplementary file 1 — Table S1: List of materials used in this study Table S2: Primer set used in this study. Figure S1: jcmm70943‐sup‐0001‐supinfo.docx. Figure S2: jcmm70943‐sup‐0001‐supinfo.docx. Figure S3: jcmm70943‐sup‐0001‐supinfo.docx. Figure S4: jcmm70943‐sup‐0001‐supinfo.docx. Figure S5: jcmm70943‐sup‐0001‐supinfo.docx. [file JCMM-29-e70943-s001.docx]

**Role of TLR4 in Enteric Glia Response to *Clostridioides difficile* Toxins: Insights from *in vivo* and *in vitro* Studies**

Maria Lucianny Lima Barbosa^¹#^, Deiziane Viana da Silva Costa^²#^*, Dvison de Melo Pacífico^¹^, Conceição da Silva Martins Rebouças¹, Cirle Alcantara Warren^²^, Renata Ferreira de Carvalho Leitão^¹^*, Gerly Anne de Castro Brito^¹^

^1^Núcleo de Microscopia e Processamento de Imagens (NEMPI), Departament of Morphology, Federal University of Ceará, Fortaleza, 60416-030, Brazil.

²Division of Infectious Diseases and International Health, University of Virginia, Charlottesville, 22908-1340, Virginia, VA, United States

**#**The authors contributed equally to this work

**Correspondence:** Dra. Renata Ferreira de Carvalho Leitão, Departament of Morphology, Federal University of Ceará, 60430-170, Fortaleza, Brazil. E-mail: [renata.carvalho@ufc.br](mailto:renata.carvalho@ufc.br)

Dra. Deiziane Viana da Silva Costa, University of Virginia Division of Infectious Diseases and International Health 200 Jeanette Lancaster Way, Charlottesville, VA, US 22903. E-mail: [deiziane2009@gmail.com](mailto:deiziane2009@gmail.com).

**Table S1: List of materials used in this study**

| **MATERIAL** | **CATALOG NUMBER** | **MANUFACTURER** |
| --- | --- | --- |
| **Immunohistochemistry** | - |  |
| Target Retrieval Solution, Low pH | K8005 | Dako |
| Hydrogen peroxide (H2O2) | Ab64218 | Abcam |
| Anti-TLR4 antibody | ab22048 | Abcam |
| EnVision FLEX, High pH, HRP | K8000 | Dako |
| Diaminobenzidine-H202 (DAB) | K3468 | Dako |
| **Cell culture** | - |  |
| Eagle’s medium modified by Dulbecco (DMEM) | 11965092 | Gibco |
| Fetal bovine serum | A5256701 | Gibco |
| Antibiotics penicillin and streptomycin | 15140-122 | Gibco |
| Sodium pyruvate | 11360070 | Gibco |
| Trypsin-EDTA | 15090046 | Gibco |
| TLR4-C34 | 18512 | Cayman |
| Opti-MEM reduced serum medium | 31985-062 | Gibco |
| Lipofectamine RNAiMAX reagent | 13778-075 | Invitrogen |
| siRNA TLR4 (s131044) | 4390771 | Invitrogen |
| siRNA control | AM4611 | Invitrogen |
| **Immunocytochemistry** | - |  |
| Paraformaldehyde (PFA) | 43388 | Alfa Aesar |
| Citrate pH 6.0 | K80004 | Dako |
| Peroxidase blockade | K80002 | Dako |
| EnVision FLEX, High pH, HRP | K8000 | Dako |
| Anti-TLR4 antibody | 482300 | Invitrogen |
| DAB | K3468 | Dako |
| Hematoxylin | K8008/K801 | Dako |
| Faramount | P36931 | Dako |
| **Immunofluorescence** |  |  |
| Paraformaldehyde (PFA) | 43388 | Alfa Aesar |
| PBS | A59065BA | Thermo fisher |
| Triton X-100 | T8787 | Sigma-Aldrich |
| Bovine serum albumin (BSA) | A2153-100 | Sigma-Aldrich |
| Anti-TLR4 | 482300 | Invitrogen |
| Anti-NFϏB-p65 | ab16502 | Abcam |
| Anti-TNF-α | ab6671 | Abcam |
| Anti-cleaved caspase-3 | AB3623 | Sigma-Aldrich |
| Anti-TLR4 (siRNA experiments) | ab22048 | Abcam |
| Anti-cleaved caspase-3 (siRNA experiments) | ab32042 | Abcam |
| Anti- TNF-α (siRNA experiments) | sc1350 | Santa Cruz Biotechnology |
| Alexa Fluor 594 | S11227 | Invitrogen |
| Alexa Fluor 488 | A11008 | Thermo Scientific |
| Donkey anti-rabbit Alexa Fluor488 (siRNA experiments) | ab150073 | Abcam |
| Donkey anti-Goat Alexa Fluor 488 (siRNA experiments) | ab150129 | Abcam |
| Donkey anti-mouse Alexa Fluor 488 (siRNA experiments) | Ab150108 | Abcam |
| ProLong Gold antifade with DAPI | P36931 | Thermo Scientific |
| VECTASHIELD HardSet antifade mounting medium (siRNA experiments) | H-1400-10 | Vector Laboratories |
| DAPI | D1306 | Invitrogen |
| Fluorescence microscopy | LM10 | Confocal Zeiss |
| **Quantitative real-time PCR** |  |  |
| RNeasy Plus Mini Kit | 74104 | Qiagen |
| High-capacity cDNA reverse transcription Kit | 4368814 | Invitrogen |
| DNase I (siRNA experiments) | 18068-015 | Invitrogen |
| iScript cDNA synthesis kit (siRNA experiments) | 1708891 | Bio-Rad |
| iTaq master mix (siRNA experiments) | 1725121 | Bio-Rad |
| StepOne Plus | 4376592 | Applied biosystems |
| **Western blotting** |  |  |
| RIPA Lysis buffer | 89900 | Thermo Scientific |
| Protease inhibitor | P8340 | Sigma-Aldrich |
| NuPAGE Bis-Tris Mini Protein Gels, 4–12% | NP0322BOX | Invitrogen |
| Bicinchoninic Acid (BCA protein Kit) | 23225 | Thermo Scientific |
| Laemmli sample buffer | 1610747 | Bio-Rad |
| β-mercaptoethanol | 1610710 | Bio-Rad |
| PVDF membrane | 1620177 | Bio-Rad |
| Anti-TLR4 | 482300 | Invitrogen |
| Tween 20 | P1379 | Sigma-Aldrich |
| Enhanced chemiluminescence -ECL | 1705060 | BioRad |
| **Realtime-glo annexin V apoptosis**  **assay** |  |  |
| Realtime-Glo annexin V apoptosis assay | JA1000 | Promega |
| Tecan Multimode Reader | - | Tecan |
| **Caspase 3/7 assay** |  |  |
| Caspase-Glo 3/7 assay system | G8091 | Promega |
| Tecan Multimode Reader | - | Tecan |

**Table S2:** Primer set used in this study.

| **Forward** | **Reverse** |
| --- | --- |
| **GAPDH:** AGAACATCATCCCTGCATCC | CACATTGGGGGTAGGAACAC |
| **TLR4:** GTTGCAGAAAATGCCAGGATG | CAGGGATTCAAGCTTCCTGGT |
| **IL-6:** GCCAGAGTCATTCAGAGCAATA | GTTGGATGGTCTTGGTCCTTAG |

**Figure S1**

**
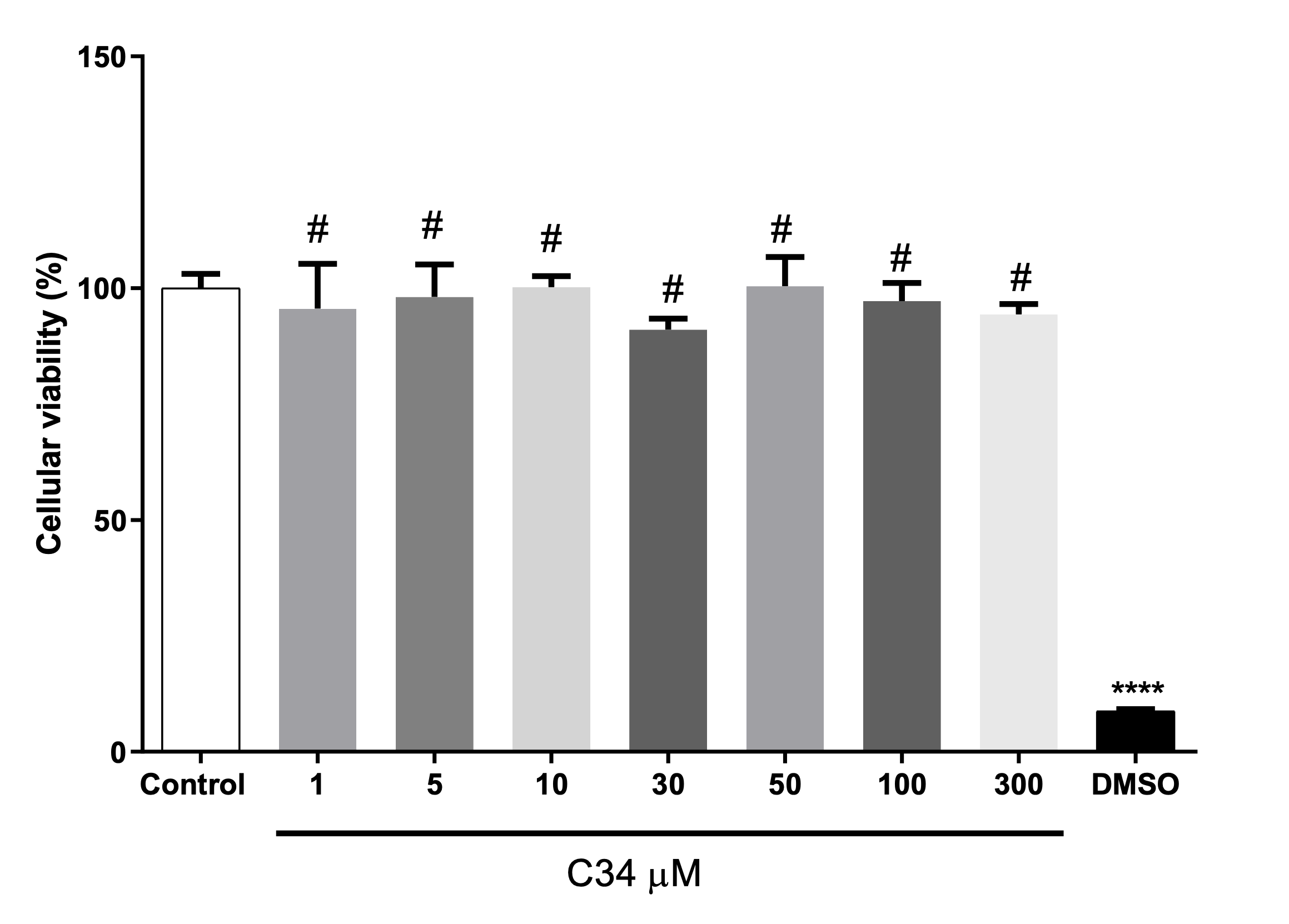
**

The viability of EGCs was assessed by MTT assay after 18h incubation with C34, a TLR4 antagonist. DMSO was used as a killing control. Data were presented as mean ± S.E.M. ****p<0.0001 versus control group (vehicle) and # not significantly different versus control group (vehicle). One-way ANOVA followed by Tukey's test was used.

**Figure S2**

**
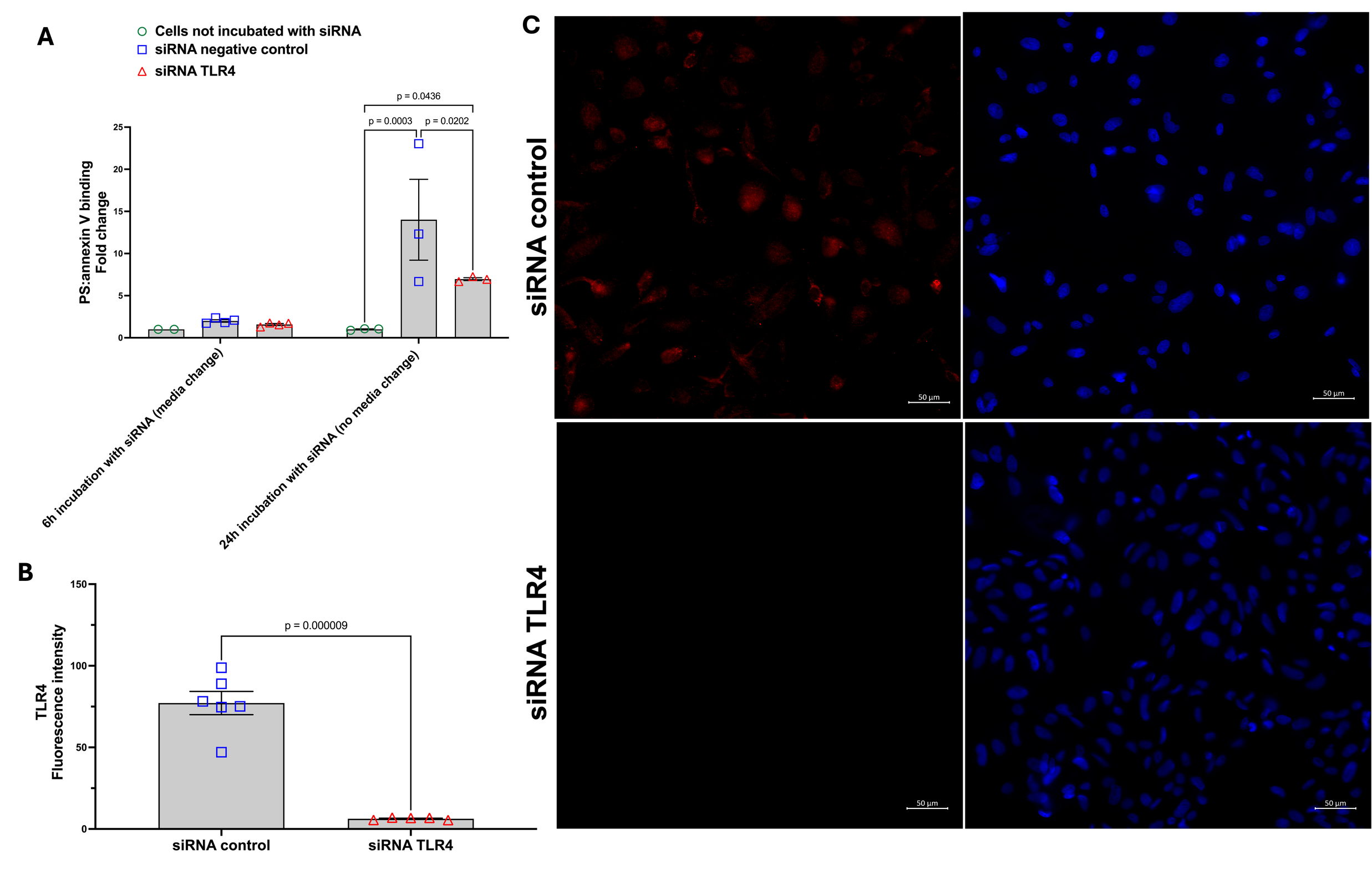
**

**A.** Cell death was analyzed by RealTime-Glo annexin V apoptosis assay in EGCs transfected with siRNA control or siRNA TLR4 for 6h followed by media replacement or for 24h without media replacement. Non-transfected EGCs were added as a control. Two-way ANOVA test was used for statistical analysis, followed by the Holm-Sidak test; The corresponding p-values are indicated in the graph. **B.** The intensity of TLR4 staining in EGCs transfected with siRNA control or siRNA TLR4 for 6h followed by media replacement and evaluated after 42h of transfection. **C.** Representative photomicrographs illustrating TLR4 (red) immunostaining and DAPI (blue) nuclear staining in EGCs transfected with siRNA control or siRNA TLR4 for 6h followed by media replacement and evaluated after 42h of transfection.

**Figure S3**


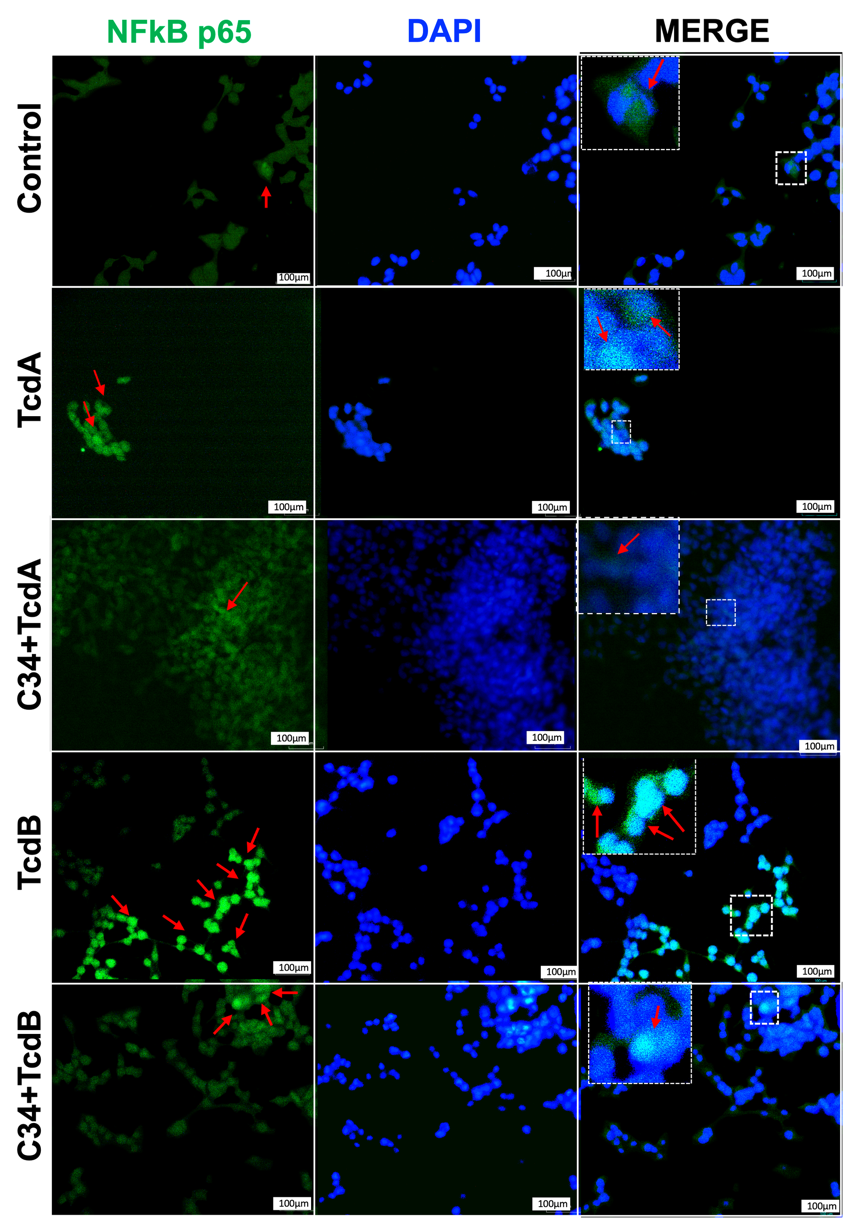


**TLR4 antagonist decreases NFkB-p65 nuclear translocation induced by TcdA and TcdB.** Representative photomicrographs illustrating NFkB-p65 (green) immunostaining and DAPI (blue) nuclear staining in EGCs exposed to TcdA (50 ng/mL) or TcdB (1 ng/mL) after 18 hours of incubation, C34 (50 µM). The nuclear translocation is indicated (arrows) by the green fluorescence (Alexa fluor 488). Merge represents the combined image of NFkB-p65 fluorescence and nuclear staining.

**Figure S4**


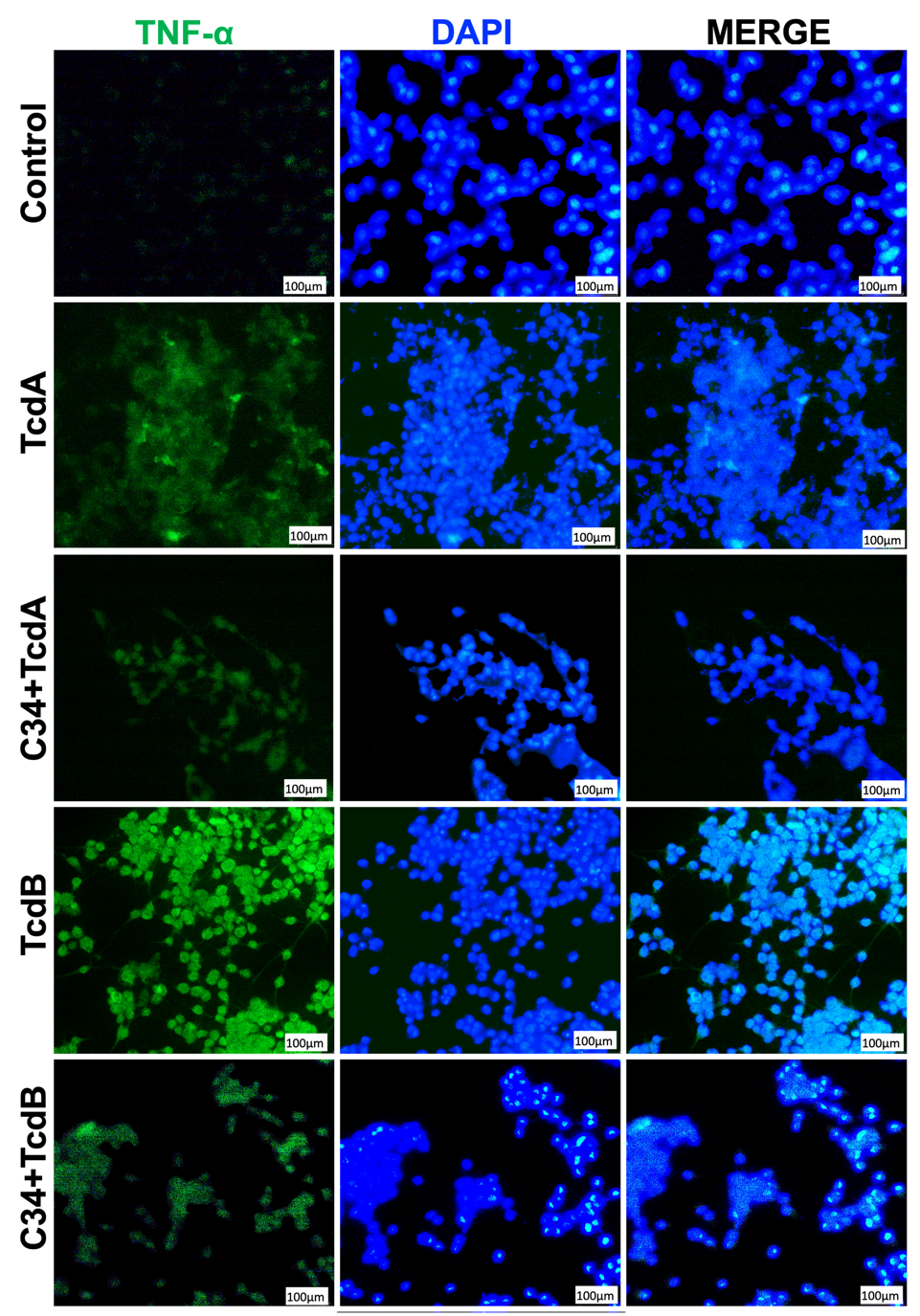


**TLR4 antagonist prevents increased TNF-α expression in EGCs induced by TcdA and TcdB**. Representative photomicrographs of TNF-α (green) immunostaining and DAPI (blue) nuclear staining in EGCs exposed to TcdA (50 ng/mL) or TcdB (1 ng/mL) after 18 h of incubation, C34 (50 µM). Merge represents the combined image of TNF-α and nuclear staining.

**Figure S5**

**
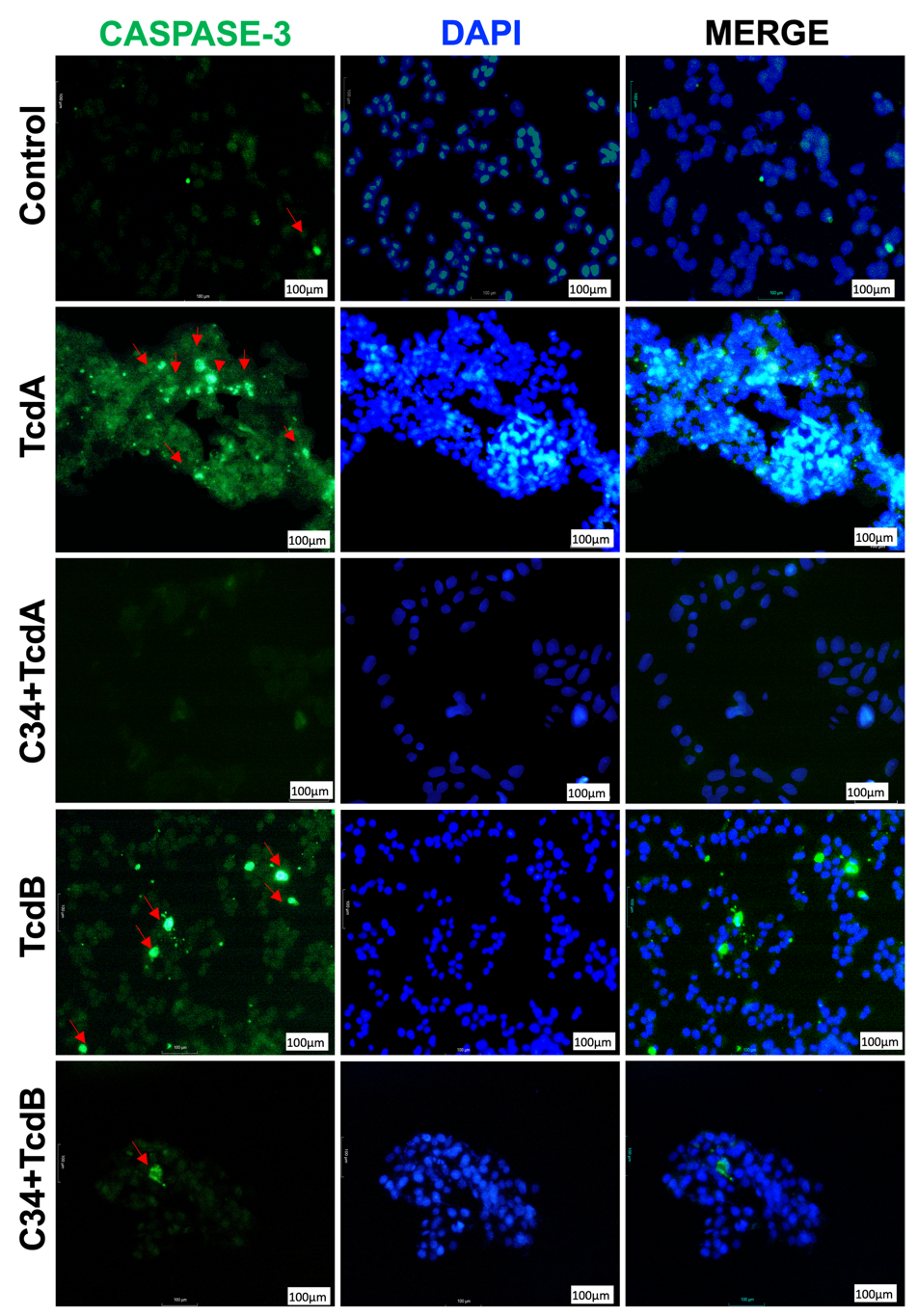
**

**TLR4 antagonist decreases cleaved caspase 3 in enteric glia exposed to TcdA or TcdB.** Representative photomicrographs of cleaved caspase-3 (green) immunostaining and DAPI (blue) nuclear staining in EGCs exposed to TcdA (50 ng/mL) or TcdB (1 ng/mL) for 18 h, Merge represents the combined image of cleaved caspase-3 fluorescence and nuclear staining, C34 (50 µM).
